# An Insight into Differentially Expressed Genes and MicroRNAs in the Pituitary Glands of the Two Estrous Phases of Sheep with Different *FecB* Genotypes

**DOI:** 10.3390/ani15030392

**Published:** 2025-01-30

**Authors:** Yue Zhang, Xiaoyun He, Ran Di, Xiangyu Wang, Mingxing Chu

**Affiliations:** State Key Laboratory of Animal Biotech Breeding, Institute of Animal Science, Chinese Academy of Agricultural Sciences, Beijing 100193, China; yueoozhang@126.com (Y.Z.); hexiaoyun@caas.cn (X.H.); diran@caas.cn (R.D.)

**Keywords:** *FecB* mutation, small tail han sheep, miRNA-seq, pituitary gland

## Abstract

As an important class of non-coding RNAs, miRNAs participate in a variety of biological processes, including cell proliferation, differentiation, apoptosis, and hormone secretion. However, little is known about the role of pituitary miRNAs in follicular development in *FecB* mutant (Fecundity Booroola) sheep. Given the critical role of the pituitary gland in follicular development, this study employed miRNA-seq technology to analyze pituitary transcriptome expression patterns during the follicular phase (F) and the luteal phase (L) of FecB mutant homozygous (BB) and wild-type (WW) of Small Tail Han sheep. This study identified differentially expressed miRNAs (DEMs) related to re-production. Of these, 10, 4, 10, and 4 were differentially expressed (DE) between BB_F and BB_L, WW_F and WW_L, BB_F and WW_F, and BB_L and WW_L, respectively. Bioinformatics analysis revealed the enrichment of the target genes of these DEMs in multiple GO terms linked to animal re-productive processes and KEGG signaling pathways. The follicular phase and luteal phase show significant enrichment in multiple pathways closely related to cell communication and signal transduction, including the MAPK signaling pathway, neuroactive ligand–receptor interaction, and the cAMP signaling pathway. In comparisons of genotypes, the calcium signaling pathway, the cAMP signaling pathway, and the MAPK signaling pathway are enriched. The miRNA-mRNA co-expression network indicates that novel121 and oar-miR-10b may regulate *CUL4B* and *ZFAND5*, respectively, playing crucial roles in sheep reproduction. Dual luciferase reporter gene assays hinted at a potential targeting relationship between novel-121 and *DNMT3A*. These findings elucidate the impact of pituitary miRNAs on follicular development influenced by *FecB* gene mutation, providing valuable insights into sheep breeding.

## Simple Summary

This study focused on the role of pituitary miRNAs in follicular development in *FecB* (Fecundity Booroola) mutant sheep. Using miRNA-seq technology, the pituitary transcriptome expression patterns during the follicular phase (F) and luteal phase (L) of *FecB* mutant homozygous (BB) and wild-type (WW) Small Tail Han sheep were analyzed. Differentially expressed miRNAs (DEMs) related to reproduction were identified. Bioinformatics analysis revealed that these DEMs’ target genes were enriched in GO terms and KEGG pathways linked to animal reproductive processes. The miRNA-mRNA co-expression network suggested that novel121 and oar-miR-10b regulate *CUL4B* and *ZFAND5*, respectively. Dual luciferase reporter gene assays hinted at a potential targeting relationship between novel-121 and *DNMT3A*. These findings provide valuable insights into the impact of pituitary miRNAs on follicular development influenced by *FecB* gene mutation, aiding sheep breeding efforts.

## 1. Introduction

The ovulation rate and the number of lambs are major reproductive traits of high economic value in sheep farming. These traits are not only influenced by environmental factors but are also profoundly regulated by genetic factors [1,2]. As a key link in the life cycle of sheep, the optimization of breeding performance has always been a hot topic in animal husbandry. The discovery of the FecB mutation among genetic factors offers a new approach to improving sheep fertility. Bone morphogenetic protein receptor type 1B (BMPR1B), also known as *FecB*, mutations were initially identified in Australian Booroola Merino sheep, a breed that has attracted much attention for its outstanding high fecundity [3]. The results showed that the *FecB* gene had an additive effect on ovulation rate, especially on the number of lambs [4], which was also verified in Small Tail Han sheep [5]. In particular, the ovulation rate and number of lambs of *FecB* mutant homozygotes were significantly higher than that of the *FecB* wild type, which further emphasizes the importance of the *FecB* gene in improving the reproductive performance of sheep [6,7]. The Small Tail Han sheep, a breed of sheep renowned for its high reproductive performance, is closely associated with the presence of the *FecB* mutation in terms of its superior reproductive capabilities [8]. Our previous study revealed a significant association between the number of lambs and the *FecB* genotype in high-yielding Chinese Small Tail Han sheep (*FecB* mutant) [5], which provided an important basis for improving the reproductive performance of sheep through molecular breeding.

The reproductive performance of sheep is not determined by a single genetic factor. As the core component of the female reproductive system, the ovaries play a crucial role in the reproductive process of sheep [9,10]. The hypothalamic–pituitary–gonadal axis regulates the reproductive endocrine system, with the pituitary gland playing a key role in follicular development and ovulation [11]. Previous studies have observed that *FecB* mutant ewes regulate their expression levels of the genes *BMPR1B*, *BMP15*, and *GDF9*, which are associated with hormone and signal transduction [12]. This regulation encompasses follicle development, ovulation, and corpus luteum formation. Studies have shown that *FecB* mutations can significantly alter the secretion pattern of LH and FSH in the pituitary gland [5], and by changing the secretion pattern of gonadotropins, it directly affects ovarian activity and thus reproductive outcomes [13].

In recent years, with the in-depth study of miRNAs, their regulatory role in the reproductive process has gradually garnered attention. As an important class of non-coding RNAs, miRNAs regulate gene expression at the post-transcriptional level by binding to the 3′-UTR of target genes. They are involved in a variety of biological processes, including cell proliferation, differentiation, apoptosis, and hormone secretion [14,15,16]. In mammals, numerous studies have demonstrated that specific miRNAs can directly or indirectly affect the function of the pituitary gland, thereby regulating reproductive performance. Specific miRNAs such as miR-488, which reduces FSH synthesis by inhibiting FSH-β gene expression [17]; miR-186-5p [18] and miR-7a-5p [19], which directly target *FSHb* to regulate FSH secretion; and miR-200b, which promotes LH production by inhibiting *ZEB1* expression, have been identified [20]. Comparative analysis of miRNA expression in the endometrium and thyroid of sheep has identified oar-miR-370-3p as a possible regulator of the *COL4A3* gene [21], further supporting the role of miRNAs in regulating reproductive processes in sheep. Since the pituitary gland plays a crucial role in regulating follicular development and ovulation, miRNAs may regulate genes within it, thereby affecting reproductive differences.

In this study, we conducted a systematic analysis of miRNA expression profiles in the pituitary gland of Small Tail Han sheep with different *FecB* genotypes across two estrus stages, employing miRNA-seq technology. Concurrently, we utilized pituitary mRNA data from the same cohort of sheep to predict and construct miRNA-target gene networks, aiming to elucidate the role of pituitary miRNAs in influencing follicular development under the regulatory effects of *FecB* gene mutations. These identified miRNAs and their corresponding target genes offer valuable insights for investigating the functional roles of the pituitary gland in follicular development.

## 2. Materials and Methods

### 2.1. Ethics Statement

The ewes participating in this experiment were all supported by the Department of Scientific Research, Institute of Animal Science, Chinese Academy of Agricultural Sciences (IAS-CAAS). In addition, the ethical approval complies with the IAS-CAAS Committee on Animal Ethics (no. IAS 2019-49).

### 2.2. Animals and Sample Collection

Using TaqMan genotyping technology [22], we genotyped the core group of Small Tail Han sheep in Yuncheng, Shandong, for selecting BB and WW genotypes ewes. Twelve healthy, non-pregnant ewes (six BB and six WW) were then randomly selected. The ewes were 33–36 months old and of similar weight. The body weights and ovulation data of these ewes are presented in Supplementary Table S1 [5]. Then, these ewes were translocated to Tianjin (117.2° E, 39.13° N) and were fed the same diet and water ad libitum. The feed formulation is shown in Supplementary Table S2.

The synchronization treatment comprised inserting a vaginal sponge impregnated with 300 mg of progesterone (supplied by InterAg Ltd., Hamilton, New Zealand), accompanied by an injection containing 50,000 IU of vitamin A and 25,000 IU of vitamin D to safeguard the vaginal epithelium [23]. Following synchronization, rams were used for teasing to assess estrus, and endoscopy was employed to observe ovarian status and confirm successful estrus synchronization.

**Figure 1:**
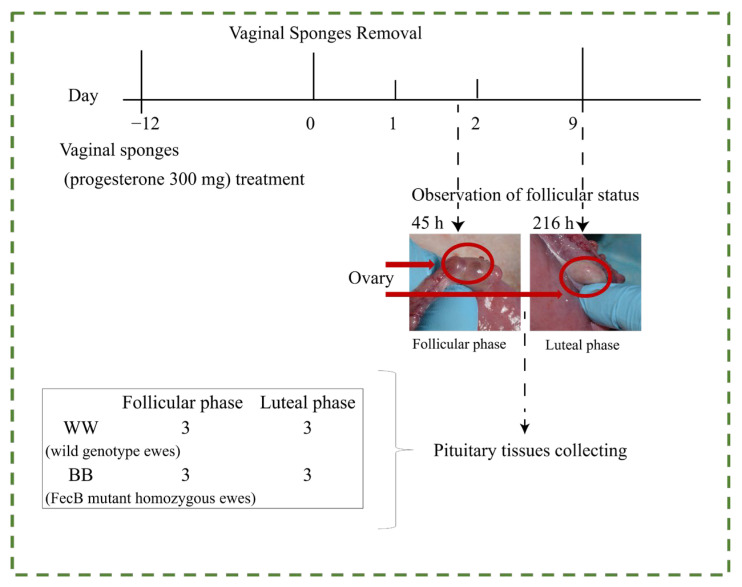
miRNA-Seq experimental design for estrus synchronization and pituitary collection in Small Tail Han sheep. The red area circled is the ovary.

Twelve days after estrus synchronization, the sponges were removed, marking this time point as baseline (0 h) (Figure 1). According to previous research on reproductive characteristics [5,7], six ewes (three BB and three WW) were euthanized 45 h after sponge removal (follicular phase, F); another six ewes (three BB and three WW) were euthanized at 216 h (luteal phase, L). Immediately following euthanasia, pituitary glands were excised [24], rapidly frozen in liquid nitrogen, and stored at −80 °C for subsequent analysis.

### 2.3. RNA Extraction, Library Construction, and Sequencing

A total of 3 μg of the total RNA was used as the starting material for miRNA library construction. Ribo-Zero™ Gold Kits (Epicentre, Madison, WI, USA) were used to remove ribosomal RNA (rRNA) from the total RNA. Twelve RNA sequencing libraries were constructed for paired-end sequencing according to the instructions of the NEB Next Ultra Directed RNA Library Preparation Kit (NEB, Ipswich, MA, USA) for Illumina platforms. Finally, the products were purified by the AMPure XP system, and an Agilent Bioanalyzer 2100 system (Agilent Co., Ltd., Beijing, China) was used to assess the library quality. The Illumina HiSeq platform was used to sequence libraries.

### 2.4. Sequencing Data Filtering and Comparative Analysis

Raw data of miRNA-Seq were stored in FASTQ file format which contains sequence information and corresponding sequencing quality information. The raw sequencing data are called raw tags. The following steps were employed: (1) reads without 3′adapter were removed; (2) reads without insert fragment were removed; (3) reads with too much poly A/T were removed; (4) reads with a length out of a certain range were removed; (5) the low quality reads were removed; and (6) the reads which contained an N base of more than 5% for total bases were removed. After filtering, the clean tags were mapped to the reference genome of Oar_v4.0 (https://www.ncbi.nlm.nih.gov/assembly/GCF_000298735.2, accessed on 10 September 2017) and miRbase21.0 (http://www.mirbase.org, accessed on 10 September 2017) with Bowtie1.0.1 [25]. miRDeep2 (https://github.com/rajewsky-lab/mirdeep2, accessed on 1 November 2017) was used to predict novel miRNA by exploring the secondary structure [26]. The known miRNAs were described with “oar-miR-”. Novel miRNAs were described with “novel-”.

We utilized the OmicShare tool (website: https://www.omicshare.com/tools, accessed on 6 January 2025) to calculate the Pearson correlation scores between miRNA samples and to generate corresponding graphs [27].

HISAT2 (v.2.0.5, http://daehwankimlab.github.io/hisat2/, accessed on 25 June 2024) and String Tie (v.1.3.2d, https://ccb.jhu.edu/software/stringtie/, accessed on 25 June 2024) were used to align and assembly the mRNA data from our team’s previous experiments (PRJNA782215) with the sheep reference genome (Oar4.0: GCF_000298735.2). The mRNA data were obtained from the same sheep pituitary source as the miRNA data. Subsequently, we employed HTSeq (v0.6.1, http://WWw–huber.embl.de/HTSeq, accessed on 14 September 2024) to calculate the read counts.

### 2.5. miRNA Targets Prediction

For animals, miRanda (version 3.3a) was used to predict targets of both the known and novel miRNAs. [28]. The principle of miRanda prediction is based on sequence alignment within the seed region. The results were filtered using the parameters -sc 160 and -en -20.

### 2.6. Analysis of DE mRNA and miRNA

We conducted differential expression analysis on both miRNA and mRNA data using the DESeq2 R package (version 4.4.1) [5,26]. In our analysis, we adopted a corrected *p*-value ≤ 0.05 and a fold change > 2 as the default thresholds for determining significant differential expression [29]. We established four comparison groups: follicular phase versus luteal phase in BB-genotype ewes (BB_F vs. BB_L), follicular phase versus luteal phase in WW-genotype ewes (WW_F vs. WW_L), follicular phase in BB-genotype ewes versus follicular phase in WW-genotype ewes (BB_F vs. WW_F), and luteal phase in BB-genotype ewes versus luteal phase in WW-genotype ewes (BB_L vs. WW_L).

### 2.7. Perform GO and KEGG Analysis on Predicted Differential Target Genes of miRNAs

To reveal the potential roles of DEMs, we performed Gene Ontology (GO) category [30] and Kyoto Encyclopedia of Genes and Genomes (KEGG) pathway enrichment analyses on their target genes using the clusterProfiler package (v.3.16.0) with the false discovery rate (FDR) padjustMethod. A *q*-value < 0.05 and fold change > 2 (log2foldchange > 1)  were set as the thresholds for significant DEMs by default [29,31].

### 2.8. Reverse Transcription (RT)-qPCR Validation

DEMs were selected randomly and RT-qPCR was used to verify the accuracy of the sequencing data. A miRNA 1st Strand cDNA Synthesis Kit (TaKaRa, Tokyo, Japan) and miRNA Universal SYBR qPCR Master Mix (Vazyme, Nanjing, China) were used. U6 small nuclear RNA (snRNA) was used as an internal reference for normalizing miRNA expression levels. All experiments were performed in triplicate to ensure reliability. RT-qPCR reactions were carried out on a LightCycler 480II (Roche, Basel, Switzerland). The PCR program consisted of initial denaturation at 95 °C for 5 s, followed by denaturation at 95 °C for 5 s, annealing at 60 °C for 30 s, extension at 72 °C for 5 s, and storage at 4 °C. The miRNA primers were designed with specific tail sequences added to their ends, and miRNA Design V1.01 and Primer 5.0 were used to design primers. The specific primers were synthesized by Shanghai Sangong Biotechnology Company (Table 1), and universal primers provided by the miRNA 1st Strand cDNA Synthesis Kit were used for miRNAs. Relative expression levels were determined using the 2^−ΔΔCt^ method [32]. Between the RT-qPCR results and the CPM values from miRNA-seq, the correlation was implemented using the Pearson correlation method of SPSS 24.0.

### 2.9. Dual-Luciferase Reporter Assays

**Figure 2:**
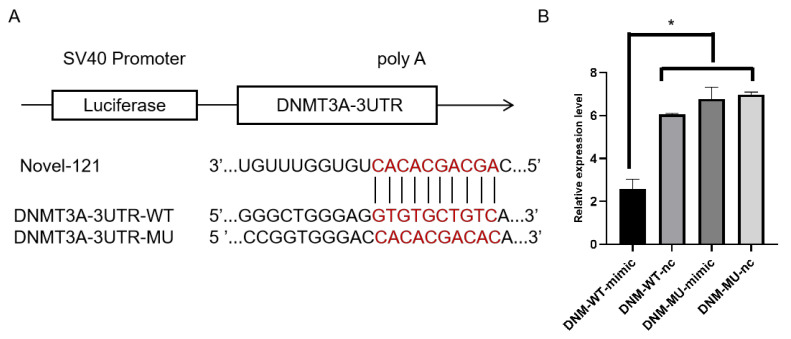
The detection of interactions between novel-121 and *DNMT3A* by the dual luciferase reporter system. (**A**) The predicted interaction between novel-121 and *DNMT3A*. The wild-type sequence GTGTGCTGTC within the 3′UTR was mutated to CACACGACAC in the mutant version. (**B**) The miR-140 mimic was cotranfected with DNMT3A 3’UTR WT or DNMT3A 3’UTR MUT to detect the luciferase activity. * *p* < 0.05.

The target relationship between *DNMT3A* and Novel_121 was verified. The wild-type 3′UTR of DNMT3A mRNA was constructed as a dual luciferase vector at the Xho I enzyme cleavage site. The fragments were inserted into the pmirGLO vector, Finally, the DNMT3A-3′UTR wild-type vector (DNM-WT) and DNMT3A-3′UTR mutant vector (DNM-MU) were successfully constructed. Specifically, the wild-type sequence GTGTGCTGTC within the 3′UTR was mutated to CACACGACAC in the mutant version. The sequences, including the wild-type/mutant sequences and miRNA mimic, illustrated in Figure 2A, were synthesized by GenePharma (Beijing, China).

The HEK293T cell line was utilized to determine the target relationship between miRNAs and their target genes. Cells were plated in 24-well plates and grown to 70% or greater confluence before transfection. Cotransfection with a DNM-WT target or a DNM-MU target and an miRNA mimic or a mimic-NC was performed with Lipofectamine 2000 (Invitrogen, CLD, USA). Then, the luciferase activities were measured using the Dual Luciferase Reporter Assay System (Vazyme, Nanjing, China) at 24 h post-transfection. The assays were performed in triplicate.

### 2.10. Statistical Analysis

When comparing trends in miRNA relative expression levels with miRNA-Seq data, as well as analyzing dual-luciferase reporter gene data, the *t*-test is employed. A *p*-value < 0.05 is considered statistically significant for differences between groups. Statistical analysis and data visualization were conducted using GraphPad Prism 8.0.2 software.

## 3. Results

### 3.1. Library Sequencing and Quality Control

**Figure 3:**
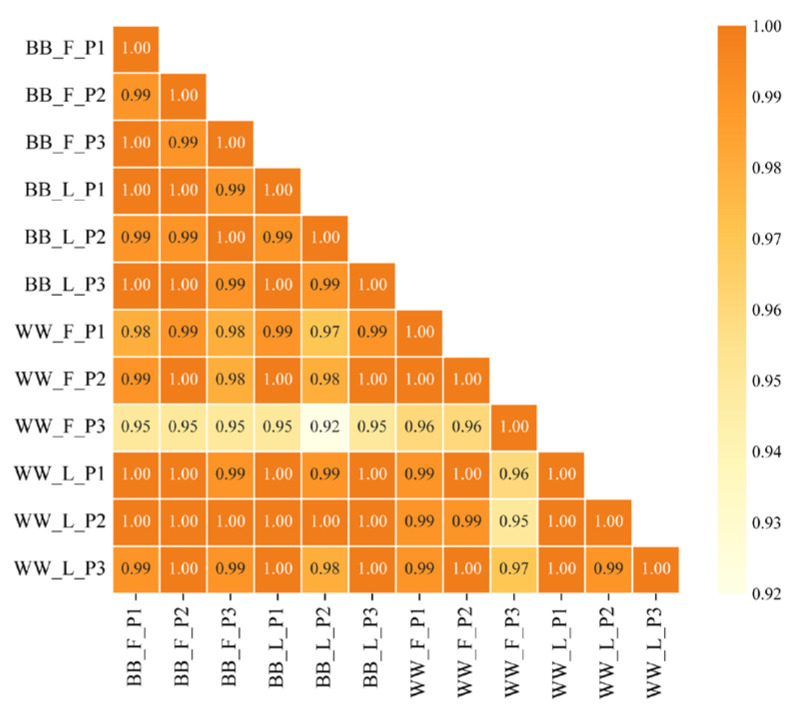
The Person correlation analysis of miRNA data among 12 samples of Small Tail Han sheep. Yellow indicates low correlation (close to −1 or 0), while orange signifies high correlation (close to 1).

Twelve small RNA libraries were obtained from the pituitary tissue of Small Tail Han sheep, and the length distribution analysis of the identified miRNA showed that most of the miRNAs were between 21 and 24 nt in length. The clean read quality scores for Q20 and Q30 were higher than 99.01% and 98.50% (Table S1), respectively, indicating that the reliability and quality of the sequencing data were sufficient for further analysis. The reproducibility and reliability of the samples were analyzed at the expression level using Pearson correlation. The correlation coefficient of miRNA expression levels between samples exceeded 0.92, indicating high consistency and reliability among samples (Figure 3). As a result, the test results of the four samples, namely BB_F, BB_L, WW_F, and WW_L, revealed the following numbers of miRNAs: BB_F identified 134 known miRNAs and 96 unknown miRNAs; BB-L contained 133 known miRNAs and 99 unknown miRNAs; WW_F included 134 known miRNAs and 125 unknown miRNAs; and WW_L had 89 known miRNAs along with 188 unknown miRNAs.

### 3.2. Screening of Differentially Expressed miRNA

**Figure 4:**
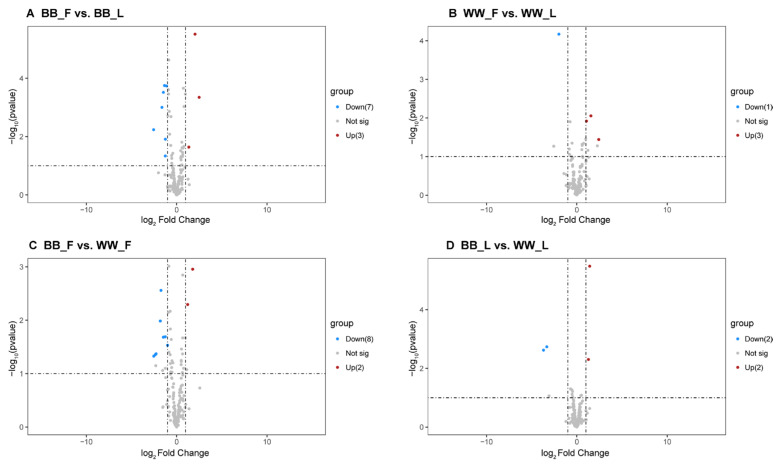
DEMs analysis among four groups. (**A**) represents the follicular and luteal phases of BB-genotype ewes (BB_F vs. BB_L), (**B**) shows the follicular and luteal phases of WW-genotype ewes (WW_F vs. WW_L), (**C**) compares the follicular phases of BB-genotype and WW-genotype ewes (BB_F vs. WW_F), and (**D**) depicts the luteal phases of BB-genotype and WW-genotype ewes (BB_L vs. WW_L). Blue and red dots represent significantly differentially expressed transcripts (*p* < 0.05) with a fold change > 2 (log2foldchange > 1), where blue indicates downregulation and red indicates upregulation. Grey dots signify transcripts that are not statistically significant (*p* > 0.05).

Comparing the expression level of miRNA in different groups of Small Tail Han sheep, with a *p*-value < 0.05 as the standard and a fold change > 2, a total of 28 miRNA were significantly differentially expressed (*p* < 0.05) (Supplementary Table S3). Among them, 10 miRNAs were significantly differentially expressed in BB_F vs. BB_L, including three up-regulated and seven down-regulated miRNAs (Figure 4A). Four miRNAs were significantly differentially expressed in the WW_F vs. WW_L group, including three up-regulated and one down-regulated (Figure 4B). Ten differentially expressed miRNAs were screened in the BB_F vs. WW_F group, including two up-regulated and eight down-regulated miRNAs (Figure 4C). Four significantly differentially expressed miRNAs were screened in the BB_L vs. WW_L group, including two up-regulated and two down-regulated miRNAs (Figure 4D). Furthermore, we conducted a differential analysis of the target genes of these differentially expressed miRNAs. The BB_F vs. BB_L comparison showed 73 differentially expressed mRNAs (36 up-regulated, 37 down-regulated). The WW_F vs. WW_L comparison showed eight differentially expressed mRNAs (four up-regulated, four down-regulated). The comparison between the BB_F and WW_F groups showed that the BB_F group exhibited 32 differentially expressed mRNAs (19 up-regulated, 13 down-regulated). Additionally, in the comparison between the BB_L group and the WW_L group, 22 mRNAs were significantly differentially expressed (15 up-regulated, seven down-regulated) (Supplementary Table S4).

### 3.3. Pathway Enrichment Analysis of miRNA Targets

**Figure 5:**
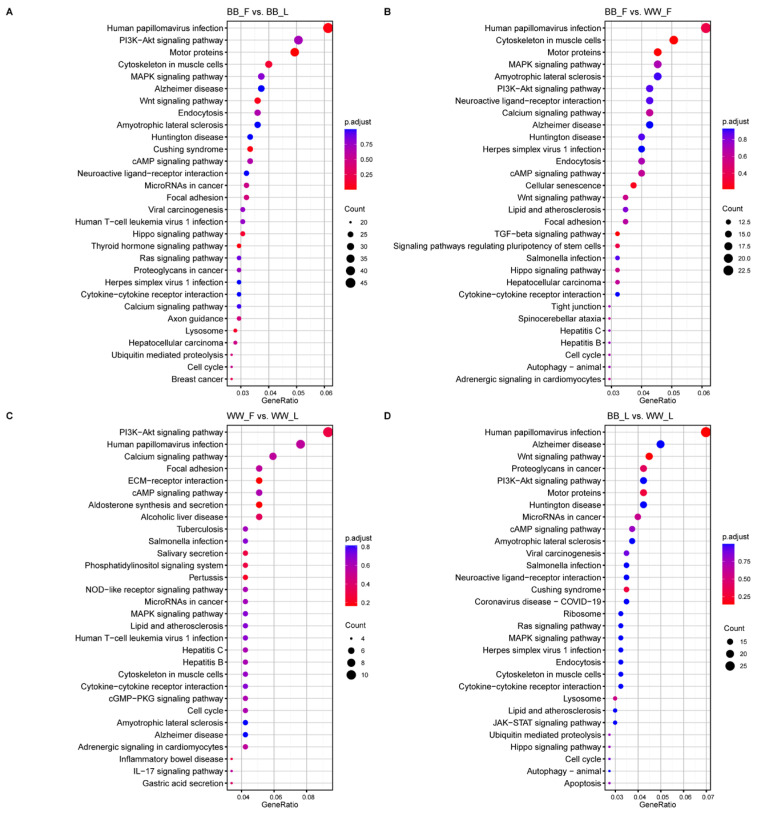
Thirty enriched KEGGs of genes targeted by DEMs in four groups. (**A**) represents the follicular and luteal phases of BB-genotype ewes (BB_F vs. BB_L), (**B**) compares the follicular phases of BB-genotype and ww-genotype ewes (BB_F vs. WW_F), (**C**) shows the follicular and luteal phases of WW-genotype ewes (WW_F vs. WW_L), and (**D**) depicts the luteal phases of BB-genotype and WW-genotype ewes (BB_L vs. WW_L). The horizontal and vertical coordinates represent the −log (*p*-value) of the enriched genes and the KEGG pathway, respectively.

Enriched GO functional terms and KEGG pathways were considered as predicted functional terms and pathways for protein-coding genes and miRNA (Supplementary Tables S5 and S6). KEGG predicted that multiple pathways closely related to cell communication and signal transduction in the follicular and luteal phases were significantly enriched (Figure 5). 

These include the MAPK signaling pathway, neuroactive ligand–receptor interaction, cytokine–cytokine receptor interaction, the cAMP signaling pathway, the calcium signaling pathway, and the TGF-beta signaling pathway. In the genotype comparison, the calcium signaling pathway, the cAMP signaling pathway, the MAPK signaling pathway, and the TGF-beta signaling pathway were enriched. In addition, pathways involved in the synthesis, secretion, and follicle–luteal transition of various hormones, including steroid hormones, growth hormones (GHs), thyroid hormones, estrogen, and insulin, were annotated in different groups.

### 3.4. Validation of RNA Sequencing Using RT-qPCR

**Figure 6:**
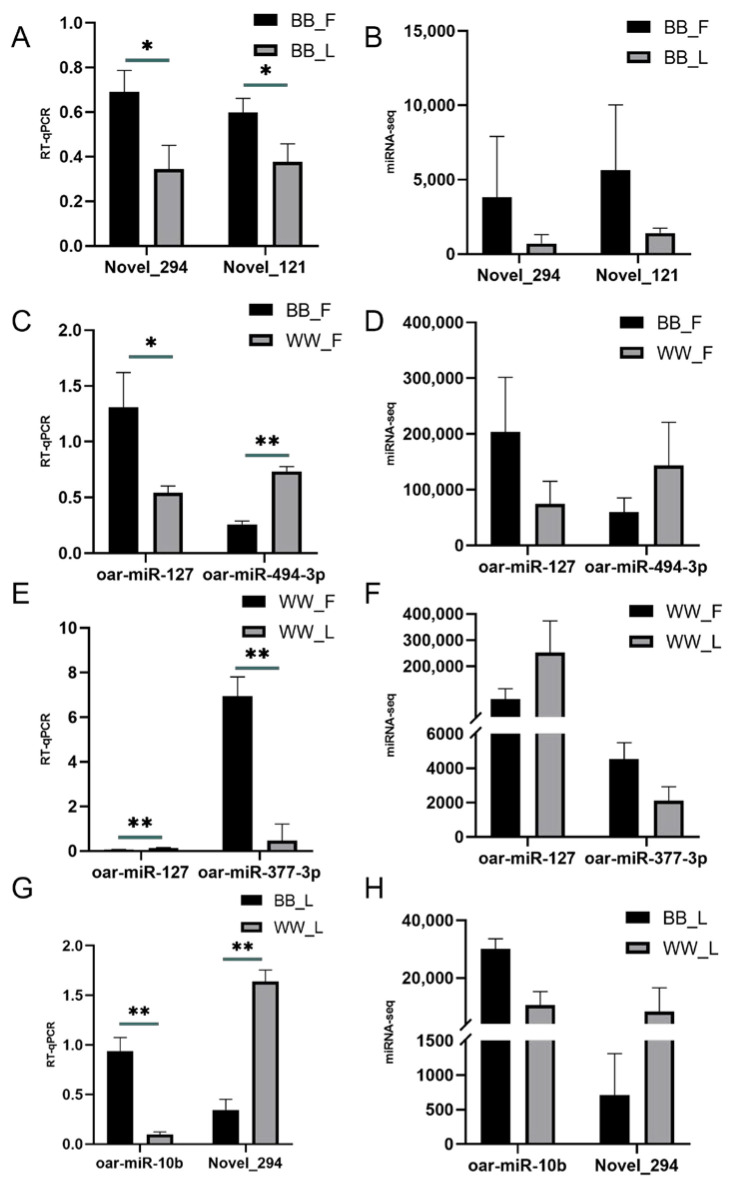
The validation of differentially expressed miRNA by RT-qPCR. RT−qPCR verified the expression trend of DEMs. The RT-qPCR data are presented as relative gene expression. miRNA-seq was the sequencing data. * *p* < 0.05 was considered statistically significant; ** *p* < 0.01 was considered particularly significant. (**A**,**B**) represent the follicular and luteal phases of BB-genotype ewes (BB_F vs. BB_L), (**C**,**D**) compare the follicular phases of BB-genotype and WW-genotype ewes (BB_F vs. WW_F), (**E**,**F**) show the follicular and luteal phases of WW-genotype ewes (WW_F vs. WW_L), and (**G**,**H**) depict the luteal phases of BB-genotype and WW-genotype ewes (BB_L vs. WW_L).

To validate the miRNA-Seq data, eight miRNAs were detected by RT-qPCR with 2^−ΔΔCt^ values (Figure 6, Supplementary Table S7). The results showed that the miRNA expression level data were reliable. Pearson correlation analysis results of all genes showed that there was a strong positive correlation between RT-qPCR and miRNA-Seq data (R^2^ ≥ 0.742, *p* < 0.05).

### 3.5. Plasmid Construction and Dual-Luciferase Experimental Validation

Dual-luciferase reporter assay indicated that novel-121 significantly suppressed the luciferase activities for co-transfecting with wild types of DNMT3A 3′UTR, while no effect on the mutant types of DNMT3A 3′UTR or blank vectors occurred (Figure 2B, Supplementary Table S8). These results initially confirmed the direct interactions between novel-121 and *DNMT3A*.

## 4. Discussion

Pituitary gonadotropins, particularly FSH and LH, play a pivotal role in reproductive activation by synthesizing and secreting these hormones at appropriate levels. *FecB* mutations have been shown to affect granulosa cells (GCs) and oocyte responses to FSH and LH during follicular development, primarily through impairing bone morphogenetic protein (BMP) signaling which leads to accelerated maturation and the ovulation of preantral follicles [33]. To gain insight into the underlying regulatory mechanisms governing the follicle–luteal transition in sheep with different *FecB* genotypes, we conducted an in-depth bioinformatics analysis of pituitary microRNAs (miRNAs) using Illumina HiSeq technology. It found that most were 21–23 nt in length, which is very similar to the size of a typical miRNA. The oar-let-7b, oar-let-7c, oar-let-7d, oar-let-7g, and oar-let-7i identified in this experiment belong to the same miRNA family. The high degree of conservation of let-7 in different animal species suggests that they may play important (and possibly similar) roles in biological processes in a variety of organisms [34,35] A study has identified miR-433 in rats and demonstrated its role in regulating the secretion of the follicle-stimulating hormone (FSH) in the pituitary [36]. This discovery echoes the potential hormonal regulatory role of miR-433 in pigs [37]. oar-miR-433 was also identified in this experiment and was found to be a differentially expressed miRNA in the pituitaries of *FecB* sheep with different genotypes. This indicates that miRNAs are conserved among species and share similar functions and mechanisms of action across different species. The oar-miR-433 identified in this experiment may also potentially have a hormonal regulatory role.

The study of miRNA in the context of *FecB* mutations in sheep yields significant insights into the transition from the pituitary to the follicle–corpus luteum. A range of differentially expressed miRNAs have been identified during the follicular and luteal phases, demonstrating their important regulatory roles. The study of miRNAs in sheep with *FecB* mutations provides key insights into the transition from the pituitary to the follicle–corpus luteum. Various miRNAs are differentially expressed during follicular and luteal phases, regulating important processes such as granulosa cell proliferation, steroid hormone synthesis, and ovarian apoptosis. The identified differentially expressed miRNAs, oar-miR-10a and oar-miR-133, play crucial roles in reproductive characteristics and neuronal survival mechanisms [38,39]. miR-299-5p may indirectly affect the release of pituitary hormones to the ovary by regulating neurons in the hypothalamic–pituitary–ovarian axis [40]. GO and KEGG analyses revealed pathways related to embryo development, cell cycle checkpoint signaling, GnRH secretion, WNT signaling, and MAPK signaling, which are vital for reproduction and fertility traits. *BDNF*, a neurotrophin family member, modulates gonadal development and cellular proliferation [41] and regulates the hypothalamic–pituitary–adrenal axis [42]. *BDNF* stimulates the MAPK-ERK1/2 pathway and may affect FSH secretion and the follicular-to-luteal transition in sheep [43]. Pituitary hormones (FSH and LH) are essential for this transition, and their synthesis and secretion involve multiple pathways, including GH, estrogen, GnRH, and folate. Regulatory elements are expected to dominate in the cell-specific expression and hormonal response of the pituitary gland. *CUL4B*, as a target gene of novel-121, exhibited a downward trend in comparison within the BBF VSBBL cohort, whereas novel-121 was correspondingly up-regulated. This observation suggests that novel-121 may function as a negative regulator of *CUL4B*, where an increase in novel-121 expression leads to a decrease in *CUL4B* expression. Mouse model studies have demonstrated that the specific knockout of *CUL4B* results in abnormal follicular development and early embryonic arrest, accompanied by reduced levels of FSH and LH [44]. This highlights the essential role of normal *CUL4B* expression in maintaining reproductive function, especially during follicular development and early embryonic stages. Consequently, it is reasonable to hypothesize that *CUL4B* must maintain an optimal level of expression during the follicular phase to ensure proper follicle development. Novel-121 may influence follicular development by regulating *CUL4B* expression during the luteal phase. Specifically, the upregulation of novel-121 may lead to the downregulation of *CUL4B* expression, which in turn may affect FSH and LH levels through an unknown mechanism. Loss-of-function *CUL4B* mutations lead to impaired cortical neurogenesis, as shown by premature cell cycle exit, precocious and unbalanced neuronal differentiation, and neuronal hyperexcitability [45]. FSH and LH are pivotal hormones that regulate granulosa cell proliferation and differentiation, as well as follicle development. This regulation may directly influence the production and release of hormones in the pituitary gland through the hypothalamus–pituitary–gonadal axis, altering the secretion patterns of FSH and LH, and thereby impacting the progression of follicular development. Novel_121, potentially regulating steroid hormone synthesis and FSH production, targets *FKBP5* and *FKBP10*, which promote pituitary steroid hormone synthesis and FSH production, respectively [46]. Another target, *DNMT3A*, a DNA methylase crucial for establishing the DNA methylation landscape in oocytes, is essential for reproduction and development [47,48]. These findings not only deepen the understanding of the complex regulatory mechanisms in sheep with different genotypes but also reveal how miRNA and its target mRNA act synergistically through these pathways to jointly regulate fecundity in sheep.

The main gene *FecB* is responsible for the characteristics of a high ovulation rate and high number of lambs in Small Tail Han sheep [3]. Through the analysis of DEGs enriched in KEGG and GO pathways, we found significant associations with multiple reproduction-related pathways, including the Hippo signaling pathway, which may regulate the secretion of luteinizing hormone in the mouse pituitary, thereby influencing the mouse reproductive process [49]. Additionally, the cGMP-PKG signaling pathway is crucial in animals [50]. Thirteen differentially expressed miRNAs, including oar-miR-10b, oar-miR-127, and oar-miR-200c, were identified in sheep with different genotypes. MiR-10b is involved in immune responses and can regulate GH levels by targeting the *SSTR2* gene in Yanbian cattle pituitary cells [51,52]. MiR-200 reduces the translational capacity of LHR mRNA by direct binding [53]. In BB sheep, the expression level of oar-miR-10b was significantly up-regulated, while its target gene, *ZFAND5*, exhibited a downward trend. As a key player in the ubiquitin–proteasome system, the downregulation of *ZFAND5* may impair the normal degradation process of proteins, particularly those involved in apoptosis and proliferation [54,55]. Importantly, *ZFAND5* functions to inhibit the activation of NF-κB [56]. Consequently, with decreased *ZFAND5* expression levels, NF-κB activation may no longer be adequately inhibited. NF-κB is a crucial transcription factor that plays a pivotal role in cell proliferation, survival, immune response, and inflammatory processes [57]. When over-activated, NF-κB can inhibit the normal apoptotic process of cells through a series of complex signaling pathways. It is speculated that the *FecB* mutation may be responsible for the differential expression of oar-miR-10b in sheep with different genotypes, which in turn inhibits the action of *ZFAND5* and activates the function of NF-κB. This inhibitory effect on apoptosis creates favorable conditions for the proliferation of pituitary cells. The pituitary gland, an important organ in the endocrine system, is responsible for the secretion of various hormones, including FSH. Under normal circumstances, FSH secretion is tightly regulated to ensure the proper functioning of the reproductive system. However, in BB-type sheep, FSH secretion or production may be accelerated due to the proliferation of pituitary cells, which may lead to functional alterations in the reproductive system. This acceleration promotes excessive follicle development in the ovary, thereby affecting the quality and quantity of eggs. Additionally, the secretion of FSH may have complex interactions with the luteinizing hormone (LH), further influencing the reproductive performance of sheep. These series of changes may collectively exert profound effects on the reproductive system of sheep.

## 5. Conclusions

In this study, multiple DEMs were found in the pituitary glands of Small Tail Han sheep with follicular- and luteal-phase FecB BB genotypes and FecB WW genotypes. These DEM genes can be significantly enriched in multiple signaling pathways and are involved in animal reproductive processes. The miRNA-mRNA co-expression network analysis showed that Novel-121 targeted *CUL4B* and oar-miR-10b targeted *ZFAND5* to regulate the NF-κB pathway, which may regulate the pituitary FSH and LH levels. By revealing the co-regulation of miRNA and miRNA in this context, our study provides valuable insights into sheep breeding.

## Figures and Tables

**Table 1:** The primer sequences designed for real-time fluorescence quantification.

Gene Name	Primer Sequences (5′-3′)	Tm (°C)
Novel_207	CTGACCTATGAATTGACAGCC	53
Novel_121	CAGCAGCACACTGTGGTTTGT	59
Novel_294	TAGCAGCACAGAAATGTTGGTA	54
oar-miR-377-3p	ATCACACAAAGGCAACTTTCGT	55
oar-miR-494-3p	TGAAACATACACGGGAAACCTCT	56
oar-miR-10b	ACCCTGTAGAACCGAATTTGTG	55
oar-miR-10a	TACCCTGTAGATCCGAATTTG	51
oar-miR-127	ATCGGATCCGTCTGAGCTTGGCT	63
U6-F	AACGCTTCACGAATTTGCGT	56
U6-R	CTCGCTTCGGCAGCACA	56

## Data Availability

The data presented in this study are available in the article.

## Supplementary Materials

The following supporting information can be downloaded at:https://www.mdpi.com/article/10.3390/ani15030392/s1, Table S1: Experimental diet composition and nutrient level (air-dried basis). Table S2: Body weights and ovulation counts. Table S3: Total set of DEMs was up- and down regulated in four groups. Table S4: Total set of DEGs was up- and down regulated in four groups. Table S5: GO enrichment of differentially expressed DEMs targets in four groups. Table S6: KEGG enrichment pathways for differentially expressed DEMs targets in four groups. Table S7: RT-qPCR data of DEMs in four groups. Table S8: Dual-luciferase report experimental data.
